# High-Strength Bolt Corrosion Fatigue Life Model and Application

**DOI:** 10.1155/2014/567318

**Published:** 2014-07-24

**Authors:** Wang Hui-li, Qin Si-feng

**Affiliations:** ^1^State Key Laboratory of Structural Analysis for Industrial Equipment, Dalian University of Technology, Dalian 116085, China; ^2^Bridge Engineering Research Institute, Dalian University of Technology, Dalian 116085, China; ^3^Research Center for Numerical Tests on Material Failure, Dalian University, Dalian 116022, China

## Abstract

The corrosion fatigue performance of high-strength bolt was studied. Based on the fracture mechanics theory and the Gerberich-Chen formula, the high-strength bolt corrosion fracture crack model and the fatigue life model were established. The high-strength bolt crack depth and the fatigue life under corrosion environment were quantitatively analyzed. The factors affecting high-strength bolt corrosion fatigue life were discussed. The result showed that the high-strength bolt corrosion fracture biggest crack depth reduces along with the material yield strength and the applied stress increases. The material yield strength was the major factor. And the high-strength bolt corrosion fatigue life reduced along with the increase of material strength, the applied stress or stress amplitude. The stress amplitude influenced the most, and the material yield strength influenced the least. Low bolt strength and a low stress amplitude level could extend high-strength bolt corrosion fatigue life.

## 1. Introduction

High-strength bolts were widely used in engineering to hold two or more parts together. Bolts and screws played an important role in the performance of machinery. The high-strength bolt connection was common in the steel structure erection joint. 1,500,000 sets of high-strength bolts were used on the Jiu Jiang Bridge [1], and more than 100 ten thousand sets of high-strength bolts were used on Chongqing Chao Tian Men Bridge [2]. The fatigue performance of high-strength bolt joint affected the bridge structure security. A review of the work done in 1943 was performed by Arnold [3]. He considered many aspects and identified heat treatment, physical dimensions, and shape of the thread form as important factors. Patterson [4] evaluated the different methods for bolt fatigue-life prediction. Burguete and Patterson [5] investigated the effect of mean stress on the fatigue limit of bolts. They compared the fatigue limits predicted using the Goodman line, the Soderberg line, and the Gerber parabola and models by Cook [6] which deal more specifically with notched specimens, with their own experimental data. Liao et al. [7] adopted Gurson-Tvergaard-Needleman (GTN) model to forecast high-strength bolt's breaking load. Zhang [8] used many kinds of analysis methods, carrying on analysis to the bolt's fracture and the substrate. Based on two-stage loose theory, Yamamoto and Kasei [9] proposed bolt and nut relative sliding quantitative model. In order to study the detention break reason during the use of high-strength bolt, Yang et al. [10] established high-strength bolt stress corrosion break mathematical model. Most studies focused on the ultimate bearing strength of the bolted joints. Xiao [11] investigated the bearing strength and failure process for double lap bolted joints under a static tensile load. Girard et al. [12] showed that the bearing strength of the bolted joint was largely depending on the clamping force. Improved numerical analysis methods were needed to reduce the expensive and time-consuming experiments.

Many steel structures were in corrosion environment. The atmosphere revealed the presence of NaCl in coastal area and revealed the presence of SO_2_ in industrial area. These factors accelerated structure corrosion. Excessive corrosion could eventually lead to a reduction in preload as parts weaken or to the total loss of clamping force through corrosion wastage or stress corrosion cracking (SCC) [13]. Many studies on corrosion fatigue had been done. However, there was no authoritative theory about it. The “wedge theory” was widely accepted [14]. The theory was that corrosion products played role as wedge in the cyclic loading process. It induced crack expansion. The fatigue life of high-strength bolt joint reduced under the coupling function of the corrosion and the fatigue load [15]. Hence, high-strength bolt's corrosion fatigue performance was worth paying attention to. The aim of the present paper was to evaluate high-strength bolt corrosion fatigue life. Based on the fracture mechanics, high-strength bolt corrosion fatigue question was analyzed. The model of the high-strength bolt corrosion fracture crack and fatigue life was established.

## 2. Stress Intensity Factor of High-Strength Bolt 

**Figure 1:**
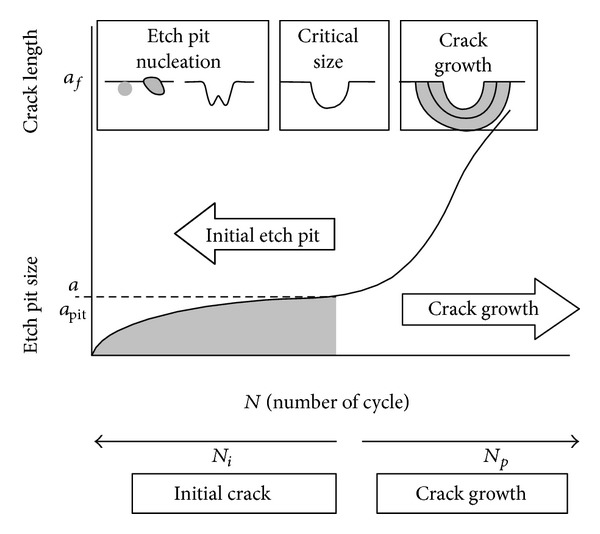
The forming and the expansion process of etch pit and the expansion process of corrosion fatigue crack.

The corrosion fatigue life of structure without the initial cracking was divided into two stages, namely, the actual life of corrosion fatigue crack and the propagation life of corrosion fatigue fracture, as shown in Figure 1.

The failure process of high-strength bolt was described by cycle number:

(1) $$\begin{array}{c} N=N_{i}+N_{p}. \end{array}$$

*N* was the total cycle number, *N*_*i*_ was initial life, and *N*_*p*_ was the crack growth life. *N*_*i*_ could be estimated with the partial strain method, and *N*_*p*_ could be estimated with the fracture mechanics method.

**Figure 2:**
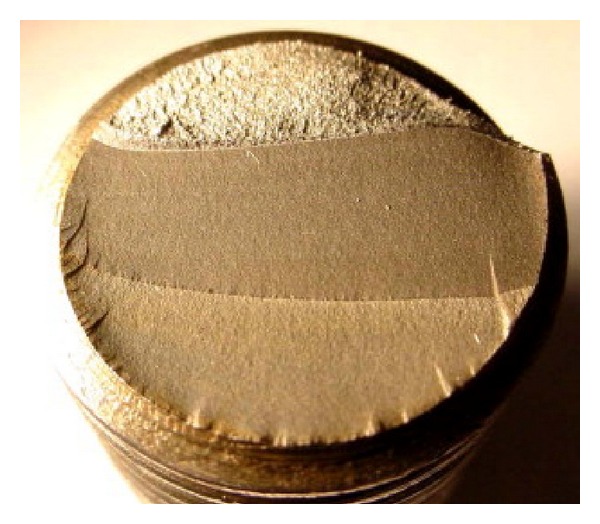
Bolt fatigue fracture.

During the production process of bolt, the micro crack occurred at the bottom of the bolt or the head rod connection. Fracture of high-strength bolt generally occurred in thread root [16], as shown in Figure 2.

The fracture mechanics holding that micro crack on the surface only started to expand if the alternating load achieves some threshold value. The stress intensity factor was calculated according to [17]

(2) $$\begin{array}{c} K_{I}=Y\sigma \sqrt{2\pi t}, \end{array}$$

where *Y* = *f*(*θ*)*F*(*d*/*D*).

In the formula *f*(*θ*) was influence coefficient of crack gap angle *θ*: $f\left(\theta \right)=\left(1-e^{-0.9(\pi -2\theta )\sqrt{D/a}}\right)/\left(1-e^{-0.9\pi \sqrt{D/a}}\right)$.

*F*(*d*/*D*) was the influence coefficient of the mutual interferes between threads:

(3) F(dD)=12[(dD)1/2+12(dD)3/2+38(dD)5/2        −0.363(dD)5/2+0.731(dD)9/2].

**Figure 3:**
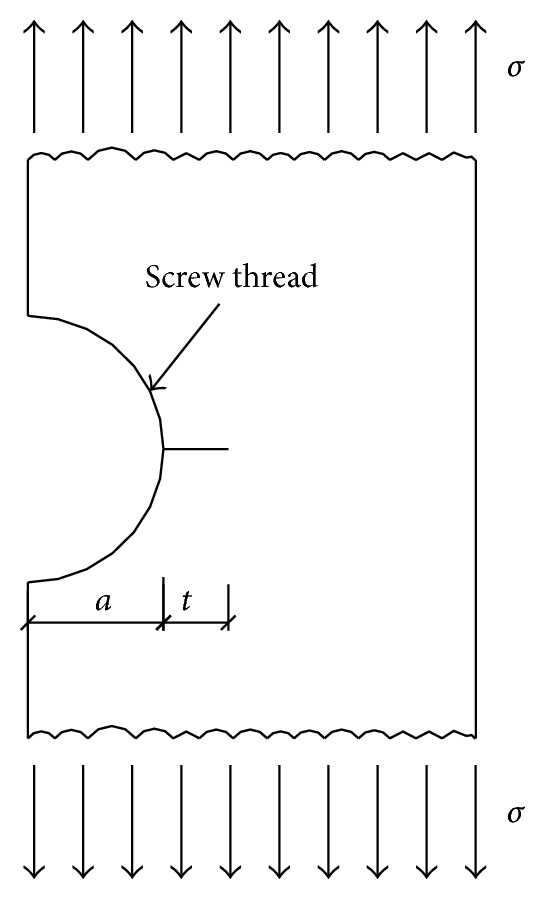
The stress diagram of bolt.

*D* and *d* were great diameter and minor diameter of the thread, *σ* was bolt applied stress, *a* was the height of thread, and *t* was the crack depth, as shown in Figure 3.

Generally, *K*_*I*_ was fit for typical plane model with penetrative crack. It belongs to two-dimensional fracture. However, the crack inside bolt belongs to three-dimensional fracture. The buried crack could be analyzed as flaky crack in infinite space. Hence, *K*_*I*_ was appropriate for bolt with pessimistic results. The inflect factor of *K*_*I*_ included applied stress and crack size. *K*_*I*_ increased with increase of applied stress. If *K*_*I*_ was big enough to expand the crack, this was critical state. *K*_*I*_ in critical state was called as fracture toughness *K*_*Ic*_. *K*_*Ic*_ was a material property, like the modulus of elasticity or the coefficient of thermal expansion:

(4) $$\begin{array}{c} K_{Ic}=Y\sigma_{c}\sqrt{2\pi t_{c}}, \end{array}$$

where *σ*_*c*_ was critical stress and *t*_*c*_ was critical crack size.

## 3. The Threshold Value of the High-Strength Bolt's Corrosion Cracking Growth

If *K*_*I*_ > *K*_*Ic*_, the crack would expand. This was fit for general environment. However, in corrosion environment, the fracture toughness should be *K*_Iscc_. *K*_Iscc_ was the threshold stress intensity factor for stress corrosion cracking. Generally, *K*_ISCC_ was much less than *K*_*Ic*_ because the corrosion environment was rich in hydrogen. Hence, the bolt was easy to fracture the corrosion environment. The high-strength bolt was often outside, corrupted by damp air and rain water. If the micro crack had existed in the thread root during the process, they would seriously reduce the crack growth life as the stress corrosion crack.

At present there was not precise expression for *K*_ISCC_. It could be estimated by Gerberich-Chen formula [18]. Gerberich-Chen believed that stress corrosion of steel was hydrogen induced cracking. They deduced Gerberich-Chen formula based on fracture mechanics:

(5) $$\begin{array}{c} K_{\text{ISCC}}=\frac{RT}{2{\bar{V}}_{H}}\ln\left(\frac{\beta }{\sigma_{s}}\right)-\frac{\sigma_{s}}{2\alpha }. \end{array}$$

In the formula,  *R* was gas constant; *R* = 8.314 × 10^−6^ MN · m/°C Mol. *T* was absolute temperature: *T* = 273 + 20 = 293 K. ${\bar{V}}_{H}$ was partial molar volume of hydrogen: ${\bar{V}}_{H}=2\times 10^{-6}\;{\text{m}}^{3}/\text{Mol}$. *α* = 13 m^−1/2^; *β* = 3170 MN*·*m. *σ*_*s*_ was material yield strength.

**Figure 4:**
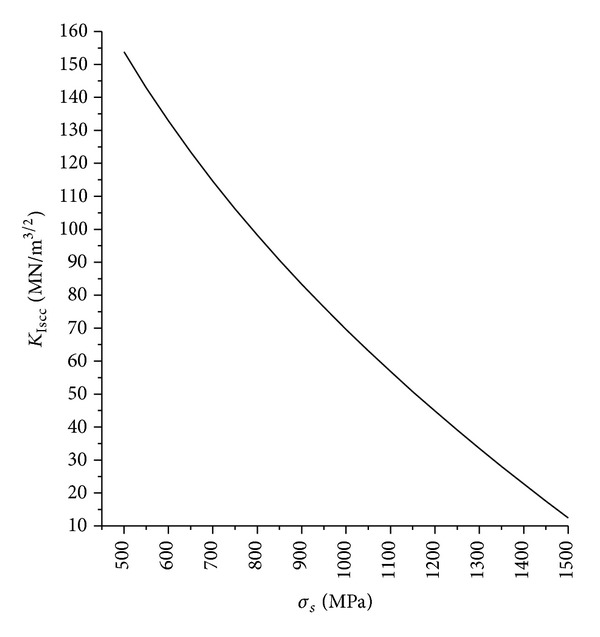
*σ*_*s*_-*K*_ISCC_ relations.

The Wellman formula showed [19] that $K_{Ic}\propto \sqrt{\sigma_{s}}$. However, according to the Gerberich-Chen formula, the threshold value *K*_ISCC_ decreased as the material yield strength *σ*_*s*_ increases. It was opposite to the Wellman formula. The *σ*_*s*_-*K*_ISCC_ relational curve was shown in Figure 4. The Gerberich-Chen formula was fit for corrosion fatigue. This had already been verified by experiments.

## 4. The Model of High-Strength Bolt Corrosion Fracture Crack

According to formula (2) and formula (4), we could get

(6) $$\begin{array}{c} K_{I}=Y\sigma \sqrt{\pi t}\leq K_{\text{ISCC}}. \end{array}$$

Then the depth of allowed crack was

(7) $$\begin{array}{c} t_{c}={\left(\frac{K_{\text{ISCC}}}{Y\sigma \sqrt{\pi }}\right)}^{2}. \end{array}$$

Substitute formula (5) into formula (7), the crack depth of the high-strength bolt corrosion fracture may be gotten:

(8) $$\begin{array}{c} t_{c}={\left(\frac{\left(RT/{\bar{V}}_{H}\right)\ln\left(\beta /\sigma_{s}\right)-\left(\sigma_{s}/\alpha \right)}{2Y\sigma \sqrt{\pi }}\right)}^{2}. \end{array}$$

## 5. The Model of the High-Strength Bolt Corrosion Fatigue Life

Paris formula [20] was commonly used to calculate the fatigue life in the fracture mechanics; namely,

(9) $$\begin{array}{c} \frac{dt}{dN}=C{\left(\Delta K\right)}^{m}, \end{array}$$

where *t* was crack size. *N* was the load cycle number. *C*, *m* was material constant. Δ*K* was stress intensity factor range.

According to the fracture mechanics theory,

(10) $$\begin{array}{c} \Delta K=Y\Delta \sigma \sqrt{\pi t}. \end{array}$$

Substituting formula (9) into (8), the result was

(11) $$\begin{array}{c} N=\frac{1}{C}\overset{a_{c}}{\underset{a_{0}}{\int }}\frac{dt}{{\left(\Delta K\right)}^{m}}=\frac{1}{C}\overset{a_{c}}{\underset{a_{0}}{\int }}\frac{dt}{{\left(Y\Delta \sigma \sqrt{\pi t}\right)}^{m}}. \end{array}$$

After integration

(12) $$\begin{array}{c} N=\{\begin{array}{cc} \frac{{t_{c}}^{1-m/2}-{t_{0}}^{1-m/2}}{C\pi^{m/2}\left(1-m/2\right){\left(Y\Delta \sigma \right)}^{m}} & m\neq 2\\ \frac{\ln t_{c}-\ln t_{0}}{C\pi {\left(Y\Delta \sigma \right)}^{2}} & m=2. \end{array} \end{array}$$

Substituting formula (8) into formula (12), the fatigue life of the high-strength bolt corrosion fracture can be estimated.

## 6. Discussion

**Figure 5:**
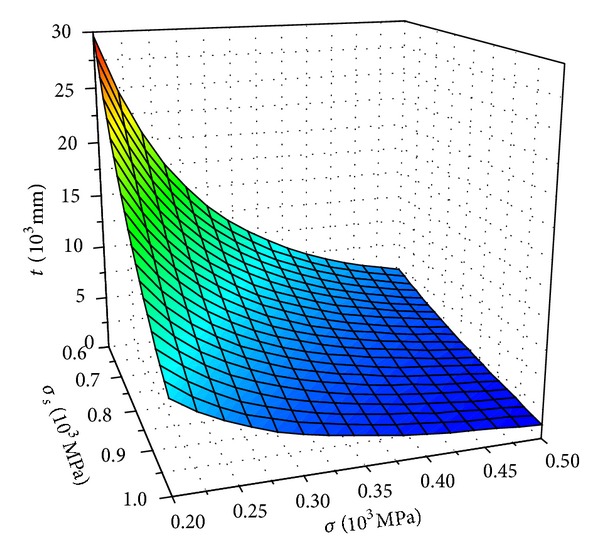
*σ*_*s*_-*σ*-*t* relations.

If *Y* ≈ 1, according to formula (2) and (6), the relations among the material yield strength, applied stress, and the allowed corrosion crack can be obtained, as shown in Figure 5.

From Figure 5, it was shown that the crack depth *t*_*c*_ of high-strength bolt was influenced by material yield strength *σ*_*s*_, applied stress *σ*. *t*_*c*_ decreased with the increase of the material yield strength *σ*_*s*_ and applied stress *σ*. The material yield strength *σ*_*s*_ had significant effect on the crack depth *t*_*c*_. Hence, the bolt strength level should be low.

**Figure 6:**
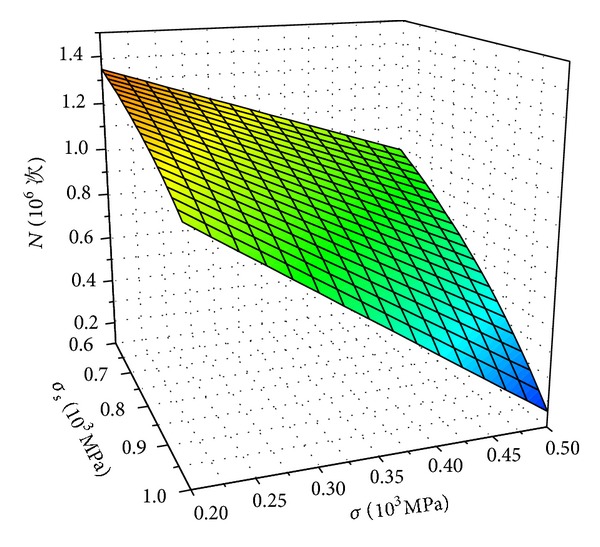
*σ*_*s*_-*σ*-*N* relations.

Moreover, suppose *m* = 3, *c* = 6.9 × 10^−12^. If *t*_0_ = 0.001 mm and Δ*σ* = 100 Mpa, according to the formula (6) and (9), the relations of the material yield strength, the applied stress, and the corrosion fracture fatigue life was obtained, as shown in Figure 6.

**Figure 7:**
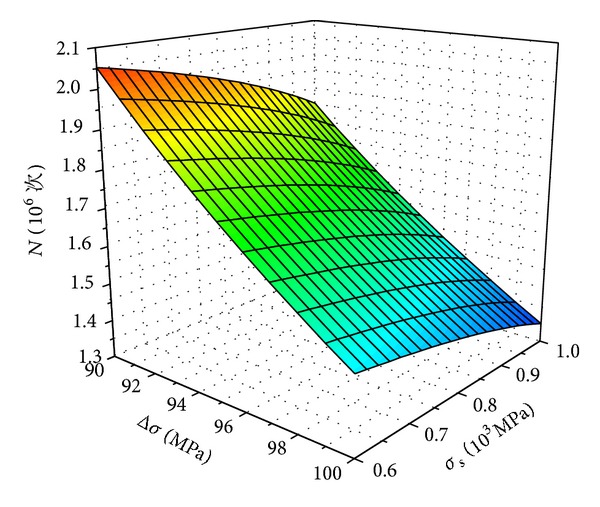
Δ*σ*-*σ*_*s*_-*N* relations.

In addition, with assumption *t*_0_ = 0.001 mm, *σ* = 100 Mpa, the relations of these factors were obtained, as shown in Figure 7.

According to Figures 6 and 7, it was found that the corrosion fatigue life of high-strength bolt was influenced by material strength *σ*_*s*_, applied stress *σ*, and stress amplitude Δ*σ*. The fatigue life reduced with the increase of material strength *σ*_*s*_, applied stress *σ*, and stress amplitude Δ*σ*. Stress amplitude Δ*σ* had significant effect on fatigue life, and the influence of material strength *σ*_*s*_ was the smallest. Hence, the stress amplitude should be controlled to a low level.

## 7. Conclusion

Based on the fracture mechanics, the corrosion fracture crack estimation model and the fatigue life estimation model of high-strength bolt were established. Then the crack depth of the high-strength bolt breaks in the corrosion environment, and fatigue life could be estimated. The factors affecting high-strength bolt corrosion fatigue life were discussed. The biggest crack depth *t*_*c*_ of the corrosion fracture high-strength bolt decreases with the increase of material yield strength *σ*_*s*_ and applied stress *σ*, in which material yield strength *σ*_*s*_ had significant effect on crack depth *t*_*c*_. Hence, the bolt strength level should be low. The fatigue life of the corrosion fracture high-strength bolt decreases with the increase of material yield strength *σ*_*s*_, applied stress *σ*, and the stress amplitude Δ*σ*. Stress amplitude Δ*σ* had significant effect on fatigue life, and the influence of material strength *σ*_*s*_ was the smallest. Hence, the stress amplitude should be controlled to a low level.
